# The Best Treatment of Cancer

**Published:** 1875-03

**Authors:** J. E. Nichols

**Affiliations:** Osage, Iowa


ARTICLE III.

### THE BEST TREATMENT OF CANCER.

BY J. E. NICHOLS, M.D., OSAGE, IOWA.

Cancer affords a large field for observation, study and careful experiment at the present day. Dr. Marsden, in his little work on this subject, says: "Only a few years since, hospital physicians and surgeons of the highest standing, as well as general practitioners, gave the poor sufferers from cancer who applied to them but one reply, 'your case is hopeless; we can be of no service to you;' and they spoke the truth."

If this is true of a few years since, what reply is made to this class of invalids by the regular profession at the present enlightened time? Whatever the reply may be, I am satisfied that enough is not done by general practitioners everywhere to save this class of unfortunates from that large list of charlatans styled "Cancer doctors." Everything going to their shops is cancer, and is sure to be *plastered* out, the merest wart being dragged out with as much sound tissue as suits their purpose, and is preserved and paraded as a terrible cancer. To give an idea of their doings let me cite the following cases:

CASE 1. Mrs. F——, of our place, aged about forty-five years, long an invalid, had, about a year ago last spring, a ligneous scirrhus, globular, of the left mamma, extirpated by the amputation of the entire left breast. It shortly returned as a "crab cancer," or radiated or branched scirrhus, and was again extirpated about a year ago the last fall. It speedily made its appearance again and extended over the entire left side of the thorax and into the axillary, her general health failing now very rapidly. While in this condition, one year ago, her husband spoke to me with regard to the employment of the "cancer paste" described on page 204, vol. 30, of the CHICAGO MEDICAL JOURNAL. I advised the use of it upon portions of the cancer, as she could bear it, and thought that if the system was properly sustained with good food and tonics, there might be some chance for improvement in her condition. But an old lady, living in a metropolitan city not a thousand miles from here, got her circulars before the patient, and the result was an immediate departure for the place of big promises. Paying her three hundred dollars in advance, and promising three hundred more as soon as the cancer was out, she had a large plaster of escharotic paste clapped on over the entire morbid growth, was informed that it would either kill the cancer or herself, and was left for forty-eight hours to the most excruciating torture that it is possible for human invention to inflict, although large quantities of whisky were administered to "deaden the pain." The side was then poulticed for several weeks, and an eschar was removed from the entire side and axillary, and she was allowed to return home, bringing the largest ulcer I ever saw upon a living human body. It gradually filled with granulations, and has steadily diminished in size from that time till now, with occasionally pain and swelling of the shoulder and arm, but with no appearance, as yet, of scirrhus.

With regard to this case, I am confident that if other means than the knife had not been resorted to, the patient would have died ere this. I also believe that a judicious use of escharotics, in the hands of a skillful surgeon, would have resulted in less pain and a more speedy cure by avoiding such extensive destruction of integument, with as perfect death to the cancer. This case establishes the fact that no limit can be fixed to human endurance.

Case 2. Mrs. B——, of an adjoining county, being similarly afflicted, and having been operated on by the knife, hearing of the above case, went to the same old lady, underwent the same torture, and returned home, to die very speedily.

Case 3. A lady of P——, Illinois, learning of Mrs. F.'s case, also went to the same woman, underwent great torture, and died a martyr to quackery.

CASE 4. Mr. J. D. S——, about thirty-five years of age, in good general health, applied to me April 14, 1874. He had a small hard point beneath the skin on the upper lip, just below the left nostril. Had been two years in forming, with occasionally pain shooting from it, and now and then a bleb or little scab appearing over it on the skin. He had lost a sister and another near relative by cancer, and I was induced to believe this an epithelial cancer, and applied the paste, before referred to, covering about one-fourth of a square inch of the integument.

April 16, I removed the paste, and ordered simple cerate dressings, which were continued six or eight days until the eschar sloughed out, and the wound had closed up with healthy granulations. I congratulated him upon so easily disposing of the source of so much uneasiness and alarm, as he had been very anxious about it for a long time. He was well pleased at first, but soon returned with the complaint that he experienced "drawing" pains and "peculiar feelings in the part," and feared it was not all out. The surface exhibited slight excoriation, but I attributed this to the action of an acrid discharge from catarrhal inflammation in the nostril. He appeared only half satisfied, and again consulted me with great alarm lest more of the cancer still remained. I advised him to rest assured that if there were still cancerous particles left, they would speedily begin to develop themselves, and that it would then be time enough to remove them. The next I heard from him was through a letter from the town of A——, in Illinois. He wrote to assure me of his every confidence in me (?) but had thought to consult those who treated nothing but cancer. He had first visited the old woman of the above cases; but becoming disgusted by her ignorance, had gone to a *he* cancer doctor, who told him that what had been done was well enough *as far as it went*, applied paste and poultices to a large part of the upper lip and produced a shocking deformity. Mr. S. returned happy in his mutilation, safe in the conviction that the cancer was now laid; but the same "drawing pains, etc.," returning, he again became alarmed, and fled to the doctor at A——. Having been paid for perfecting a cure there was no further profit in more treatment, so he told S. to rest assured that it was all right and sent him back. He is not fully at rest about it yet, and whether he will visit other cancer doctors, and pay them large figures for removing portions of his face, as long as any *face* is left, remains to be seen in the future. He no more required the last operation than he needs one now, and I am quite sure that he has no cancer at present, yet his agonies no doubt are as genuine as if possessed of true scirrhus or epithelial cancer.

While it is true that these so-called specialists pronounce every simple tumor, or ulcer, cancer, and speculate on the fears of their victims, it is none the less true that they do occasionally permanently remove true cancer. I have known of several cases, where the cancer had returned after extirpation with the knife, that they have successfully treated. People have a passion for the mysterious, and more willingly pay out their money, and risk their lives and health, for a secret medicine than for a sensible treatment that they can understand in all its details. Yet I am convinced that we have well known agents that are better for the treatment of cancer than the exclusive employment of the knife, which is so greatly feared, and is almost invariably avoided until the most favorable time for treatment is passed. If the entire profession would familiarize themselves with their use, I am satisfied that cancer doctors would be compelled to seek other employment, and possibly might become honest. With the agents to which I refer, I believe every practitioner could keep his field of labor almost, if not entirely, free from cancer cases.

The following cases are not entirely void of interest:

CASE 1. Mrs. J. B——, aged fifty years, sent for me Dec. 10th, 1873. Has been scrofulous and an invalid several years. No cancer in her family. For several months a small, hard, painful tumor has been coming on the gums of incisor teeth and roof of the mouth, obviously caused by wearing false teeth on an illy fitting rubber plate. Some five weeks before, a little-pill fellow had bunglingly endeavored to extirpate it with a pair of scissors and table fork. It had grown very rapidly from the time of that mutilation, and was now as large as a walnut. Diagnosed it to be an epithelial cancer. Treatment. — ℞. chloride zinc, pulv. blood root, *aa* q. s. Rub together in open air till it forms a stiff mass or paste. Put enough on cotton wool to cover the entire surface of the cancer, wedge more cotton wool between it and the upper lip, tell her to avoid allowing the saliva to come in contact with it as much as possible, and instruct her to remove it and have the paste wiped entirely out of the mouth when she has had it there long enough to become tired.

Dec. 11th. Patient had left the paste in position about two hours, and on examination I found a white thin eschar over the entire surface of tumor, and detached and loose in places. Pocketed some paste under the loose parts of eschar, and applied more on the surface as before. I returned in about two hours and removed it myself.

I repeated this process for ten days, each day removing detached parts of the eschar, and applying fresh paste, both by pocketing it under loose parts of the eschar and on cotton wool. At the end of this time the wound presented that freedom from cancerous particles that one soon learns to recognize in using this paste. The applications were quite painful at times, but not dreaded as would have been the knife. She said when it was all over that she would rather undergo it all over again than have the scissors employed as they had been, even for a few minutes.

During treatment I prescribed for the general health as follows: ℞. tinct. ferri. perchlor., acid. phosphor. dil., *aa* M.x ; aquæ, ℥ ij. Misce. Sig. Take it morning and night. This was followed for a few weeks with ℞. brom. potass. ʒiij, syrup simp. ℥vj, fl. ex. nux vom., fl. ex. mandrake, *aa* f. ℥ ss. Misce. Sig. Teaspoonful three times a day. There has been no return of the tumor, and good health has prevailed up to the present time.

CASE 2. Miss S——, aged twenty-eight years, deformed by hip disease and curvature of the spine. A sister had cancer. Sent for me Jan. 13th, 1874. Small, round tumor, caused by pressure of a projecting tooth, on the inside of lower lip near the margin. Very painful of late, and growing rapidly. Diagnosed it epithelial cancer. In its treatment I cut a hole in a piece of adhesive plaster, to correspond to the size of the tumor, and pressed it down around it. Put the paste on a quarter of an inch thick over the surface of the cancer, and covered all with a patch of plaster. Jan. 15, removed the plaster and paste, exposing a perfect eschar surrounded by not very extensive swelling. Declares that there has been no pain to speak of. She has suffered great pain during her life, which may account for this indifference. Directed her to keep the place covered with court plaster, which was continued about twelve days when the eschar had fallen out and the wound healed. This lady was kept on the iodide and bromide of potash for several months, and has had a good degree of health and no return of cancer up to this date.

CASE 3. Mrs. M——, mother of five children, in feeble health and a victim to the habit of opium eating. Was called to her Jan. 22, 1874. There had been an amputation of the left breast for ligneous (globular) scirrhus, by Dr. S——, some three months before my visit. It had healed kindly, but for the last month the cicatrice had been sore and swollen, with "darting pains shooting from it into the chest." On examination I discovered three points of what appeared to be scirrhous growth. Surrounded them with plaster, and applied the paste.

Jan. 28th. The eschars all out. One was two-thirds of an inch long, one-half an inch wide, and five-eighths of an inch in depth; another, one and one-half inches long, an inch wide, and an inch and one-fourth in depth. The other was between these two in size. Poultices had been used after the paste had been employed for forty-eight hours, and were continued until nearly healed, which had occurred at the end of a month. No recurrence up to date.

CASE 4. Mrs. Mary C., aged thirty-five years, in good health, called at my office Nov. 14, 1874. Has had a small, painless lump just beneath the skin, on the cheek, for the last two years. Hardly knows when it first came, and is aware of no cause. None of her relatives have ever been subject to cancer, yet she has been very uneasy of late, fearful that this is a cancer. Diagnosed it to be a simple, insignificant, little *lump* of broken-down, effete particles. Prognose that it will never amount to more than what it now is. "Can you remove it without any cutting?" is her inquiry. I reply that I can. "Will it make a bad scar?" "No." "Then please remove it," is requested, and I forthwith apply the paste, instruct her to remove it at the end of the second day, and then apply court plaster until it comes out and gets well. I saw her a couple of days ago, and observed that it was healed nicely and the cheek smooth, with a very small scar that will become much less. While any one has any little permanent tumor of that nature, which they do not understand, they suffer from the constant apprehension that it will eventuate in cancer, and if the family physician, whom they usually repose confidence in, merely poh pohs at it, they will visit a cancer doctor and pay a round price for its removal. The above case is a fair sample of over twenty that have applied to me during the last twelve months. In fact, scarcely a day goes by without some wart or diseased point, of no significance, is exhibited to me for the purpose of gaining my opinion. If they still manifest uneasiness after being assured that the troubles are harmless, I proceed to remove them. If

I was a cancer specialist I could soon amass a small fortune; as it is, I have cases come from a long distance for treatment of cancer.

CASE 5. Nils Anderson, a Swede, fifty years of age, from Forest City, sixty miles west of this place, came to my office Dec. 21, 1874. Has had a little scabby sore on his under lip the last six months; lately it has grown very rapidly, and has become very painful. It now occupies the entire under lip, is red and purple on its surface, and is seamed and squamous near its centre, discharging an acrid fluid. [I send a picture of this case, just as he came to me, to the editors of the JOURNAL]. Diagnosis, epithelial cancer. Dec. 22. Apply the paste. Dec. 23. As the paste has become dry and baked, I remove it and apply a fresh quantity. Dec. 24. Remove the paste and apply bread and milk poultices. Dec. 30. The eschar comes away this morning on removing the poultice. It is two and a half inches long, three-fourths of an inch wide, and five-eighths of an inch thick, at its greatest diameters. Some of the cancer remaining near the right angle of the mouth, I apply the following: ℞. arsenious acid, ʒij; mucilage gm. acacia, ʒj. Make into a stiff paste, apply it over surface of cancer, press a fold of lint down over it, and when it becomes hardened cut away surplus lint with scissors. This is Dr. Marsden's "Cancer Paste," and should be used on not more than one square inch of surface at one time. I like it where the greater part of a cancer has been removed, and only a point or two of it is left behind in the wound, as this paste has more of a special election for the cancer than the zinc paste, and will not destroy so much of the surrounding healthy flesh, even when used in excess. Jan. 1st. Removed this paste by soaking it off with warm water, and have applied poultices ever since. A deep line of demarcation is now formed around the remaining part of the cancer and I expect it to drop out in a day or two more. The rest of the wound, on the left half of the lip, is filled with healthy granulations, and is healing finely. I will report the result of treatment in this case at some future time.

CASE 6. Mr. F. Cole, a young man whose case I mentioned on page 206, vol. 30, of the CHICAGO MEDICAL JOURNAL, as having been before our County Medical Association at one of their meetings, with a tumor in the antrum of Highmore, with regard to which the universal verdict was that the right superior maxillary must be extirpated, and which I was then removing by the occasional application of paste through a small opening formed by the removal of several teeth, which the tumor had nearly crowded out. The tumor continued to form rapidly, as I removed it, and as Mr. C. lived some distance from my office I did not see him as often as I desired, yet in six months from the time of making the first application, it was entirely free from all morbid growth, and the plates of bone that had been crowded out, so extensively as to cause a very great deformity, had contracted very much. Now the parts have so nearly assumed their natural relation that it is only close observation that detects any deformity. Tr. of iodine and sol. of argenti nitras was used alternately, in the antrum, after the removal of every vestige of the morbid growth. A few scales of bone came away. In all this treatment he suffered not a particle of pain, and was not hindered a day from his labor, as he called on me only as he came to town on business. The result in this one case pays me for all the annoyance I have had from the employment of the paste; yet this is but a small per cent. of benefit resulting from its use during the last three years of my professional labors.

I do not advise the entire abandonment of the knife in cancer. On the contrary, I should very much dislike to undertake some cases without permission to employ it. In case of a large cancerous growth, as scirrhus of the breast, the shortest and most humane way to a cure would be, first, its extirpation with the knife; and second, the use of the paste on any new growths that would start up in the cicatrice. But in these numerous cases of imagined cancer, as well as uncertain tumors and genuine epithelial growths, I should in every instance advise the paste. While removing one of these uncertain growths, you may be nipping in the bud a very serious malady. Many times, when the knife alone is advised, the patient will object to an immediate operation, and have the treatment delayed until more positive of its malignity, and soon awaken to the knowledge that it has gone too far for successful extirpation; while, on the other hand, if the paste had been advised, the patient would not have delayed an hour, and both he and his physician might never know that his life had been so nearly endangered.

On the whole, I believe that many lives that would otherwise be sacrificed, may be saved by its timely employment.

Besides causing an eschar, the paste excites healthy inflammation in the parts around the point of application. I have taken advantage of this quality, and used it to rouse up indolent ulcers and other chronic troubles which I may think worthy of another article at some future time. Meanwhile let me ask my professional brethren to give this agent a fair trial, and let it stand or fall according to its own merits.

